# Osteogenic Differentiation of Human Mesenchymal Stem Cells Modulated by Surface Manganese Chemistry in SLA Titanium Implants

**DOI:** 10.1155/2022/5339090

**Published:** 2022-01-13

**Authors:** Jin-Woo Park, Yusuke Tsutsumi, Eui-Kyun Park

**Affiliations:** ^1^Department of Periodontology, School of Dentistry, Kyungpook National University, Daegu 41940, Republic of Korea; ^2^Research Center for Structural Materials, National Institute for Materials Science, Tsukuba 305-0047, Japan; ^3^Department of Oral Pathology and Regenerative Medicine, School of Dentistry, Kyungpook National University, Republic of Korea

## Abstract

The manganese (Mn) ion has recently been probed as a potential candidate element for the surface chemistry modification of titanium (Ti) implants in order to develop a more osteogenic surface with the expectation of taking advantage of its strong binding affinity to the integrins on bone-forming cells. However, the exact mechanism of how Mn enhances osteogenesis when introduced into the surface of Ti implants is not clearly understood. This study investigated the corrosion resistance and potential osteogenic capacity of a Mn-incorporated Ti surface as determined by electrochemical measurement and examining the behaviors of human mesenchymal stem cells (MSCs) in a clinically available sandblasted/acid-etched (SLA) oral implant surface intended for future biomedical applications. The surface that resulted from wet chemical treatment exhibited the formation of a Mn-containing nanostructured TiO_2_ anatase thin film in the SLA implant and improved corrosion resistance. The Mn-incorporated SLA surface displayed sustained Mn ion release and enhanced osteogenesis-related MSC function, which enhanced early cellular events such as spreading, focal adhesion, and mRNA expression of critical adhesion-related genes and promoted full human MSC differentiation into mature osteoblasts. Our findings indicate that surface Mn modification by wet chemical treatment is an effective approach to produce a Ti implant surface with increased osteogenic capacity through the promotion of the osteogenic differentiation of MSCs. The improved corrosion resistance of the resultant surface is yet another important benefit of being able to provide favorable osseointegration interface stability with an increased barrier effect.

## 1. Introduction

Titanium (Ti) has good corrosion resistance due to its surface oxide layer, which serves as the backbone for biocompatibility as a substrate for load-bearing metallic implants [1, 2]. However, bioinert Ti has a limitation in terms of biological activity; it does not induce active new bone formation [3]. Thus, much of researchers' efforts have been devoted to developing a more effective surface modification modality for the fabrication of Ti implants, with enhanced bone healing capacity that induces rapid new bone formation and achieves a more favorable clinical outcome, especially in poor quality bone [4–9].

Surface chemistry alteration using bioactive ions that favors early cellular events and promotes osteoblast differentiation of MSCs is a powerful approach to producing Ti implants with increased osteogenic capacity [4, 5, 7, 9]. Mn has been recently introduced as a surface modification of Ti implants [10–13] and probed as an alloying element in metallic implant substrates [14, 15]. Mn incorporation seems to favor osteogenesis-related cellular function and bone healing of implant materials [11–13, 16–18]. Mn is an essential trace element in the human body that plays a crucial role in various physiological functions by serving as a cofactor for key enzymes [19], but excessive Mn uptake causes neurotoxicity and cardiovascular dysfunction [20, 21]. Despite the possible toxicity induced by Mn overexposure through ingestion or occupational exposure, a very minute amount of Mn incorporation into the surface of Ti oral implants would not be expected to cause any adverse effects because the total amount of ion release from the oral implant would be too low to be toxic.

Studies have demonstrated that Mn has a very potent capacity for binding to integrins [22–25], binding to integrins of cells at a quite low concentration [25]. Increased integrin binding to biomaterial surface triggers integrin-mediated signaling cascades, which in turn subsequently activates favorable osteogenesis-related signaling pathways [5, 26–28]. However, the exact mechanism by which Mn enhances osteogenesis-related functions of stem cells when incorporated into the surface of Ti implants is not clearly understood. Moreover, studies have reported conflicting results on the optimal Mn concentration for inducing osteogenesis around endosseous implants [10–13, 17]. In addition, few studies have investigated the osteogenesis-related cellular functions affected by Mn modification of the Ti implant surface in human stem cells.

In addition, the capacity for corrosion resistance should be considered as another indispensable factor in the development of Ti implants with increased bone healing capacity [29–31]. A Ti surface with improved corrosion resistance would provide a more stable interface between bone tissue and the Ti implant and has been linked with long-term biological stability [29–31]. Thus, it is expected that a Ti implant surface with increased osteogenic capacity combined with improved corrosion resistance would be beneficial for a more favorable osseointegration outcome, because such a surface not only promotes early bone formation but also achieves better long-term biological stability at the bone-implant interface.

This study is aimed at investigating Mn doping to the surface of a clinically available Ti implant used in dental practice, in this study an SLA (sandblasted, large-grit, and acid-etched; Straumann AG) implant, in an effort to effect favorable osteogenesis-related functions in human MSCs. We expected the results obtained using human stem cells and commercial oral implant surface would provide significant insight into the development of surface modified Ti implants for future clinical applications. For this purpose, we incorporated Mn ions into the SLA implant surface by wet chemical treatment and then examined corrosion behavior and osteogenesis-related cell functions using primary human adipose derived multipotent stem cells.

## 2. Materials and Methods

### 2.1. Sample Preparation

To produce the SLA-type implant surface, commercially pure Ti disks (ASTM grade 4, 15 mm in diameter and 3 mm thick) were sandblasted using large-grit Al_2_O_3_ blast particles. This was followed by a double etching process using a mixed solution of H_2_SO_4_ and HCl (the SLA surface). Samples were subsequently washed in deionized water. Surface Mn modification of the SLA sample was performed by two-step wet chemical treatment, according to a method described previously [10]. Briefly, SLA samples were immersed in 5 M NaOH at 60°C for 24 h and then ultrasonically cleaned in deionized water for 30 min. After treatment, samples were further treated hydrothermally in 5 mM MnCl_2_ solution at 180°C for 4 h in a Teflon-lined reactor (the Mn-SLA surface) and then thoroughly cleaned and air dried.

### 2.2. Surface Characterization

The micro- and nanoscale surface morphologies of the investigated samples were evaluated by field emission-scanning electron microscopy (FE-SEM; S-4800, Hitachi, Tokyo, Japan). Three-dimensional surface microtopography and roughness values of the samples were determined by confocal laser scanning microscopy (CLSM, LSM700; Carl Zeiss, Oberkochen, Germany) over a 300 *μ*m × 300 *μ*m area (*n* = 5) using the LSM software (ZEN, Carl Zeiss). The crystalline structure and chemical composition of the investigated surfaces were evaluated by thin-film X-ray diffractometry (XRD; X'Pert-APD, Philips, Almelo, Netherlands) and X-ray photoelectron spectroscopy (XPS; K-Alpha; Thermo Scientific, East Grinstead, UK).

### 2.3. Mn Ion Release Measurements

Mn ion release from the Mn-SLA samples was evaluated by inductively coupled plasma-atomic emission spectroscopy (ICP-AES; 720 ICP-OES, Agilent, Santa Clara, CA, USA) under two different test conditions, i.e., a dynamic mode with gentle shaking of the samples at 50 rpm and a static mode without any shaking of the samples. Each of the five Mn-SLA sample disks was immersed in 1 mL of physiological saline solution (0.9 mass% NaCl) in a sealed bottle at 37°C with or without gentle shaking (at 50 rpm). At the indicated immersion time points, the Mn-eluted solution was retrieved and replaced with new saline solution. The concentration of Mn ions released from the Ti samples into the solution was measured after 4 h and also 1, 3, 7, and 14 days of immersion. Measurements were repeated three times for each sample.

### 2.4. Corrosion Behavior Tests: Electrochemical Measurements

The corrosion resistance capability of the SLA and Mn-SLA surfaces was tested in physiological saline (0.9 mass% NaCl aqueous solution). All of the investigated specimens were ultrasonically cleaned in ethanol for 5 min. The corrosion tests were performed under open-air condition. The electrochemical measurements were performed with a potentiostat (HABF-501G, Hokuto Denko, Tokyo, Japan) and a function generator (HB-111, Hokuto Denko). A silver-silver chloride electrode (Ag/AgCl) and a platinum black electrode were used as a reference and a counter electrode, respectively. The specimen was fixed in a specially designed polytetrafluoroethylene (PTFE) holder apparatus described elsewhere [32]. The exposed area of investigated samples contacting the electrolyte was 0.35 cm^2^ (6.7 mm in diameter), which was used as a working electrode. After immersing the specimens into the test solution at 37°C, the corrosion potential at open-circuit condition (*E*_corr_) was measured for 24 h. The potential of the Ag/AgCl electrode was calibrated with a saturated calomel electrode (SCE). The gradient anodic potential was applied at a constant sweep rate of 1 mVs^−1^ from the final value of *E*_corr_ to 1.5 V_SCE_.

### 2.5. Cell Culture Experiments

#### 2.5.1. Cell Culture

Human multipotent mesenchymal stem cells (MSCs) derived from adipose tissue (PromoCell, Heidelberg, Germany) were cultured using MSC growth medium (C-28010, PromoCell). The cells were cultured under 100% humidity and 5% CO_2_, at 37°C. The medium was changed every 3 days. After plating of the cells on Ti disks in 24-well culture plates, osteogenic differentiation was induced with MSC osteogenic differentiation medium (C-28013, PromoCell).

#### 2.5.2. Early Cell Proliferation Assay

Cells were cultured for 24 and 48 h on Ti disks in 24-well culture plates at initial seeding density of 4 × 10^4^ cells/well. Early cell proliferation was assessed by a colorimetric assay using a Cell Counting Kit-8 (CCK-8, Dojindo Molecular Technologies, Tokyo, Japan) as described previously (*n* = 7 per group) [5].

#### 2.5.3. Evaluation of Spread Cell Morphology

Cell morphology, cytoskeletal arrangement, and focal adhesion formation in adherent human MSCs on the investigated samples were evaluated at 24 and 48 h by CLSM and FE-SEM observation at an initial seeding density of 1 × 10^4^ cells/well. The distribution of focal adhesion contacts and organization of actin filaments in adherent MSCs were identified following double staining of actin and vinculin using diluted monoclonal anti-vinculin (Sigma-Aldrich, St. Louis, MO, USA), goat-anti-mouse IgG (Invitrogen, Carlsbad, CA, USA), and fluorescein isothiocyanate- (FITC-) labeled phalloidin (Sigma-Aldrich) according to a method described previously [5]. Morphology of spread cells, including cell shape and formation of attachment apparatus, on the investigated samples was further evaluated by FE-SEM. Adherent MSCs on the investigated Ti sample disks were sequentially fixed with 2% glutaraldehyde and 1% osmium tetroxide and then dehydrated using an ascending series of alcohols. After critical point drying and gold−palladium coating, the morphologies of the spread cells on the samples were observed by FE-SEM.

#### 2.5.4. Real-Time Polymerase Chain Reaction (PCR) Analysis of Adhesion- and Osteogenesis-Related Gene Expression

Human MSCs were seeded on Ti disks in 24-well culture plates at an initial seeding density of 4 × 10^4^ cells/well. We investigated mRNA expression levels of critical integrin genes (*β*1 and *β*3) and cytoskeletal adhesion proteins (RhoA, talin, and vinculin) that are known to affect subsequent osteogenesis-related cellular functions [5, 26–28, 33] at 24 and 48 h of culture.

Osteogenesis-related gene expression in human MSCs grown on the SLA and Mn-SLA samples was evaluated after 7 and 14 days of culture. The expression of critical transcription factor genes regulating osteoblast differentiation (Runx2, osterix) and osteoblast phenotype genes (type I collagen (COL), alkaline phosphatase (ALP), bone sialoprotein (BSP), osteocalcin (OC), and osteoprotegerin (OPG)) was evaluated in cells grown on the investigated samples. Reverse transcription was performed using total RNA extracted from the cells cultured on seven Ti disks, and real-time PCR was performed as described previously [5] using the primers shown in Table 1. Data were analyzed using the 2^-*ΔΔ*CT^ method, normalizing it against GAPDH. Expression levels of various genes were expressed as fold differences of the gene expression relative to the results of control SLA surface.

#### 2.5.5. Alkaline Phosphatase (ALP) Activity

To evaluate ALP activity in human MSCs grown on the samples, cells were cultured on Ti disks for 7 and 14 days (at an initial seeding density of 4 × 10^4^ cells/well). Total cellular ALP activity in the cell lysates was measured in 2-amino-2-methyl-1-propanol buffer, pH 10.3, at 37°C with *p*-nitrophenyl phosphate as the substrate. The absorbance change was measured at 405 nm using a microplate reader (*n* = 7 per group). ALP activity was expressed as nanomoles of *p*-nitrophenol (PNP) liberated per microgram of total cellular protein.

#### 2.5.6. Enzyme-Linked Immunosorbent Assay (ELISA) for the Detection of Osteocalcin Protein Production by MSCs

The protein concentration of terminal marker for osteoblast differentiation (OC) secreted by adherent cells on the investigated samples into cell culture media was measured with commercially available OC ELISA kit (R&D Systems, Minneapolis, MN, USA) at 14 and 21 days of culture (at an initial seeding density of 4 × 10^4^ cells/well). The OC protein levels in the supernatant sample of cell culture media were measured at 450 nm according to the manufacturer's instruction (*n* = 7 per group). The data were normalized to the total cellular protein content.

### 2.6. Statistical Analysis

Three independent cell culture experiments were performed. Statistical analysis was performed using nonparametric Mann–Whitney *U* test to evaluate differences between groups. *P* < 0.05 was considered statistically significant.

## 3. Results and Discussion

### 3.1. Surface Characteristics of the Samples

**Figure 1:**
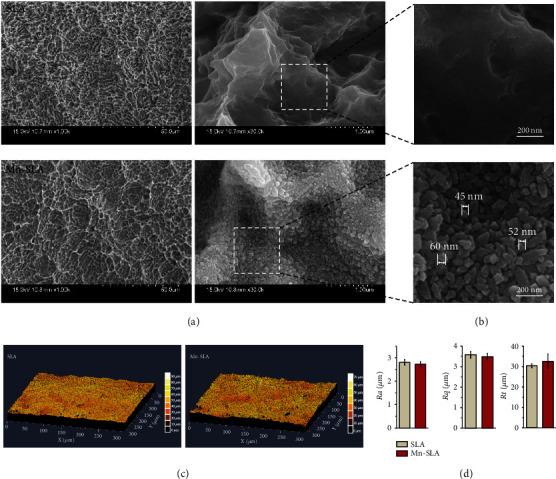
Surface characteristics of the SLA and Mn-SLA surfaces. (a) FE-SEM images of the investigated surfaces at magnifications of ×1000 and ×30,000. (b) Higher magnification FE-SEM images showing the dimension of surface nanostructures of the unmodified SLA and Mn-SLA surfaces. (c) 3D topographical measurement images by CLSM. (d) Surface roughness values determined by CLSM (Ra: average value; Rq: root mean square value; Rt: maximum peak-to-valley height value).

Figure 1 shows the surface morphology and roughness values of the investigated samples. At a low FE-SEM magnification (×1000), the SLA and Mn-SLA samples displayed almost identical surface morphologies (Figure 1(a)). Both surfaces exhibited characteristic surface morphology produced by the sandblasting/acid-etching process, i.e., fine micropits (1-2 *μ*m in dimension) superimposed on the macrorough concavities. At higher magnifications (×30,000), the SLA and Mn-SLA samples exhibited a notable difference in surface morphology at the nanometer level (Figure 1(a)). The SLA surface was smooth at the nanometer scale, whereas the Mn-SLA surface exhibited the formation of distinct surface nanostructures of approximately 50 nm in dimension (Figure 1(b)). Figures 1(c) and 1(d) are topographical measurement images and the surface roughness values of the investigated samples determined by CLSM. Similar to the FE-SEM observation results, there were no notable differences in the 3D surface microtopographies or roughness values between the two surfaces. The average surface roughness values (Ra) of the SLA and Mn-SLA samples were 2.81 ± 0.12 *μ*m and 2.73 ± 0.11 *μ*m, respectively (Figure 1(d)). These findings indicate that wet chemical treatment produces uniform surface nanostructures without any alteration of the original micron-scale surface features of the SLA implant.

**Figure 2:**
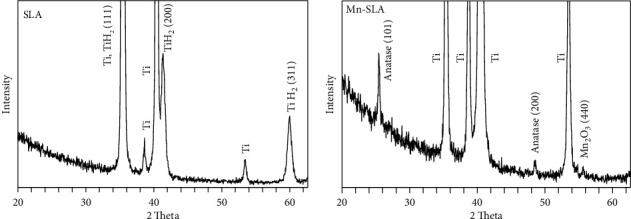
Thin-film X-ray diffraction patterns of the SLA and Mn-SLA samples.

Thin-film XRD patterns of the investigated samples are shown in Figure 2. The SLA sample exhibited the presence of titanium hydride (*δ*-TiH_2_, JCPDS #25-982), which is a typical feature found on the surface of acid-etched Ti implants [34, 35]. In contrast, the Mn-SLA surface did not display TiH_2_ peaks, which indicates the wet chemical treatment leads to desorption of hydrogen from the surface oxide layer of the SLA sample. Wet chemical treatment using Mn-containing solution leads to the formation of a Mn-containing crystalline TiO_2_ composite thin film on the surface of the SLA sample. The Mn-SLA sample exhibited the formation of an anatase TiO_2_ structure (JCPDS #21-1272) and a small peak of manganese oxide (Mn_2_O_3_; JCPDS #78-0390).

The chemical compositions of the SLA and Mn-SLA samples determined by XPS analysis are shown in Table 2. A small amount of aluminum (Al) was detected in both the SLA and Mn-SLA samples, which is attributable to the minute Al_2_O_3_ grit remnants present on the implant surface after the sandblasting process. Carbon (C), nitrogen (N), and sodium (Na) were detected as contaminants on both surfaces. The Mn-SLA sample had a surface Mn content of 2.9%.

**Figure 3:**
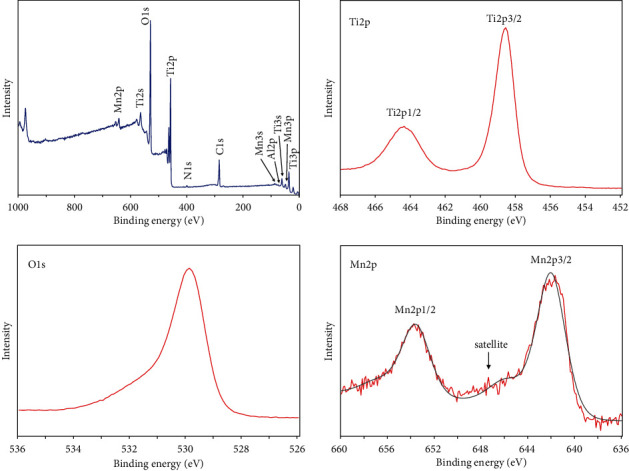
X-ray photoelectron survey spectra and high-resolution spectra of Ti2p, O1s, and Mn2p of the Mn-SLA sample.

The XPS survey spectra and high-resolution spectra of Ti2p, O1s, and Mn2p of the Mn-SLA sample are shown in Figure 3. The value of 285.0 eV for the C1s line was used as a charge correction reference. In the Ti2p region of the Mn-SLA sample, Ti2p_3/2_ and Ti2p_1/2_ were detected at 458.6 and 464.4 eV, respectively. The Ti2p_3/2_ peak at 458.6 eV and binding energy splitting of 2p_1/2_–2p_3/2_ (Δ = 5.8 eV) are attributed to the Ti^4+^ chemical state of TiO_2_ [36, 37] The binding energy of the O1s level in the Mn-SLA sample was 530 eV, which is typical for anhydrous oxide, the O^2–^ state [37, 38]. Mn2p peaks were observed at 641.8 eV (Mn2p_3/2_) and 653.4 eV (Mn2p_1/2_), which correspond with the binding energies of Mn_2_O_3_ [39]. In addition, a weak shoulder structure was observed between the Mn2p_3/2_ and Mn2p_1/2_ peaks, which is attributable to a small satellite peak present at around 647.4 eV. This finding may indicate the additional presence of a Mn^2+^ state [40]. Thus, the Mn in the thin surface film of the Mn-SLA sample appears to exist in two oxidation states, Mn^2+^ and Mn^3+^.

### 3.2. Mn Ion Release Determination

**Figure 4:**
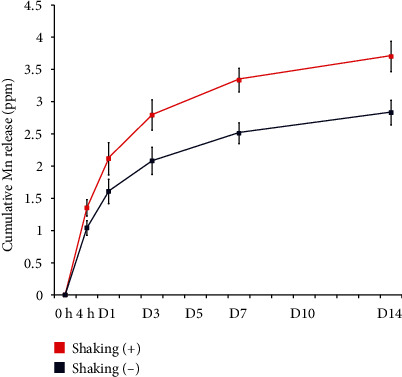
Release of Mn ions from the Mn-SLA sample under two different test conditions (with or without shaking of the samples) as determined by inductively coupled plasma-atomic emission spectroscopy (ICP-AES) analysis. Data are presented as the mean ± SD (*n* = 5).

Figure 4 shows the concentrations of Mn ions released from the Mn-SLA samples into saline solution under two different test conditions, as determined by ICP-AES analysis. The Mn-SLA surface displayed sustained ion release. In the dynamic mode (gentle shaking of samples at 50 rpm), the Mn-SLA sample released a higher amount of Mn ions than in the static mode (without any shaking of the samples). The Mn-SLA sample released a relatively high amount of Mn ions into saline solution upon 3 days of incubation (2.79 ± 0.24 ppm in the dynamic mode and 2.08 ± 0.21 ppm in the static mode), and this decreased over time. Mn concentrations upon 24 h of immersion were 1.61 ± 0.18 ppm for the static mode and 2.11 ± 0.25 ppm for the dynamic mode. The cumulative total concentrations of Mn ions released from the Mn-SLA samples in the dynamic and static modes for 14 days of incubation were 3.7 ± 0.23 ppm and 2.83 ± 0.19 ppm, respectively. These results indicate that the Mn-containing TiO_2_ layer obtained by wet chemical treatment can serve as an effective platform for the delivery of Mn ions to adherent cells on the SLA implant surface.

### 3.3. Corrosion Behavior of the Samples as Determined by Electrochemical Tests

**Figure 5:**
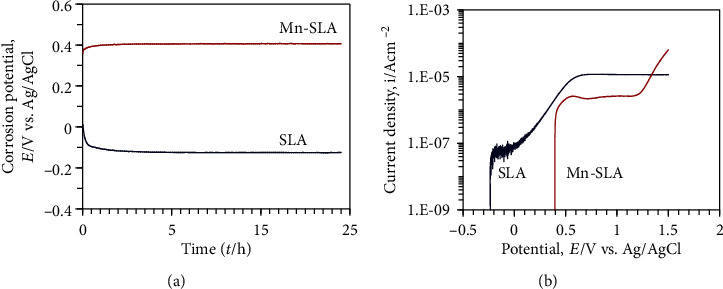
Corrosion behavior of the investigated samples determined by electrochemical measurement. (a) Open-circuit potential and (b) polarization curves of the samples tested in physiological saline solution.

The corrosion potential of the Mn-SLA sample during 24 h immersion in physiological saline was much higher than that of the SLA sample (Figure 5(a)). In addition, the value of the corrosion potential of the Mn-SLA sample was stable. It suggests that the surface oxide layer formed on the Mn-SLA sample drastically inhibits the corrosion reaction and that this can be maintained even after immersion in physiological saline for 24 h.

The polarization curve of the Mn-SLA surface also displayed a unique pattern (Figure 5(b)). The change in the current density of the Mn-SLA surface at around the time of the initial stage of the experiment was steep because the corrosion potential of the Mn-SLA surface was much higher than that of the SLA surface. However, the Mn-SLA surface was passive until 1.2 V (Ag/AgCl) and the passive current density of the Mn-SLA surface was smaller than that of the SLA surface. Therefore, it appears that the corrosion resistance of the Mn-SLA sample is much better than that of the SLA sample. These findings indicate that the barrier effect against the corrosion reaction through the passive film can be improved by wet chemical treatment to incorporate Mn ions in the SLA implant.

It has been reported that nanostructured anatase TiO_2_ coatings obtained by hydrothermal treatment improve corrosion resistance of Ti-based implants including pure Ti and Ti13Nb13Zr [41]. In this study, a two-step wet chemical treatment produced a Mn-containing film along with a crystalline anatase TiO_2_ structure in the SLA implant surface. We reported in a previous study employing the same treatment conditions as in the present study that the thin Mn-containing TiO_2_ film produced by wet chemical treatment was approximately 0.5 *μ*m thick [10]. Thus, the increased corrosion resistance of the Mn-SLA sample is attributable to the dense surface oxide layer composed of an anatase TiO_2_ structure along with the formation of crystalline Mn_2_O_3_ structure. Therefore, it is expected that Mn-incorporating process using wet chemical treatment can be utilized as a surface treatment modality which can improve the corrosion resistance of microstructured Ti implants.

### 3.4. The Early Human MSC Response Affected by Surface Mn Modification

#### 3.4.1. Early Cell Proliferation and mRNA Expression of Adhesion-Related Genes

**Figure 6:**
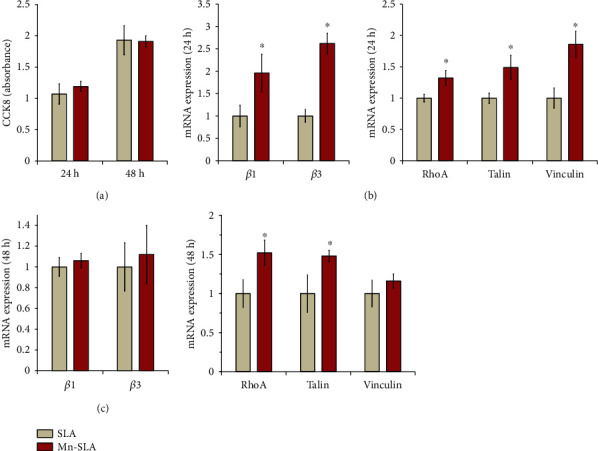
Early response of primary human mesenchymal stem cells to the investigated surfaces. (a) Early cell proliferation expressed as absorbance value at 24 and 48 h of incubation. Quantitative real-time polymerase chain reaction analysis of the levels of mRNA for *β* integrin genes (*β*1 and *β*3), RhoA, talin, and vinculin in cells grown on the investigated surfaces at (b) 24 h and (c) 48 h of culture. Values are the mean ± SD of three independent experiments. ^∗^*P* < 0.05 between two surfaces.

There was no difference in early human stem cell proliferation (expressed as the absorbance value of CCK-8) between the SLA and Mn-SLA surfaces at 24 and 48 h of culture (Figure 6(a)). We investigated whether surface Mn modification would affect the mRNA expression of adhesion-related genes of human MSCs when introduced into an SLA implant. Notable differences were found in the mRNA expression levels of critical adhesion-related genes of MSCs between the two investigated samples (Figures 6(b) and 6(c)). At 24 h of incubation, the expression of the *β*1 and *β*3 integrin genes was upregulated in cells grown on the Mn-SLA surface compared with those on the SLA surface (*P* < 0.05; Figure 6(b)). In addition, mRNA expression levels of RhoA, talin, and vinculin, which are known to play central roles during the cell adhesion process [5, 9, 42], were significantly increased in cells grown on the Mn-SLA surface compared with those on the SLA surface (*P* < 0.05; Figure 6(b)). At 48 h of incubation, there were no differences in the mRNA expression levels of the *β*1 and *β*3 integrin subunits between the SLA and Mn-SLA surfaces (Figure 6(c)). The Mn-SLA surface still supported a higher level of RhoA and talin mRNA expression compared with the SLA surface at 48 h (*P* < 0.05; Figure 6(c)).

Studies have demonstrated that overexpression of the *β*1 and *β*3 integrins in bone-forming cells subsequently induces favorable osteoblast differentiation in both the micro- and nanostructured Ti implant surfaces [5, 33, 43]. Vinculin, talin, and RhoA are cytoskeletal proteins, which act as essential regulators of integrin-mediated cell adhesion and link integrins to the actin cytoskeleton [5, 33, 42, 44]. Thus, the expression mode of these cytoskeletal adhesion proteins regulates integrin-mediated intracellular signaling and also contributes to the subsequent osteogenesis-related signaling pathways of MSCs. Considering the mRNA expression patterns of the early adhesion-related genes in this study, Mn incorporation induces more favorable early cellular events that are required for subsequent osteogenesis-related functions in anchorage-dependent osteogenic cells on the implant surface.

#### 3.4.2. The Spread Cell Morphology and Focal Adhesion Development of MSCs

**Figure 7:**
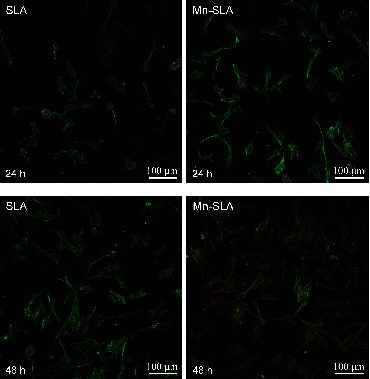
Merged CLSM images of the spread human mesenchymal stem cells on the investigated surfaces showing actin cytoskeleton (green) and focal adhesions (red) at 24 and 48 h of incubation.

Figure 7 shows the morphologies of human MSC spread on the SLA and Mn-SLA samples evaluated by CLSM observation. The Mn-incorporated SLA surface supported better cell spreading compared with the control SLA surface at both 24 and 48 h of incubation time. At 24 h, cells grown on the SLA surface exhibited relatively weak cytoplasmic extensions. In contrast, cells on the Mn-SLA surface showed a more spread morphology with extensive cytoplasmic extensions. At 48 h, human MSCs grown on both surfaces displayed a more spread appearance, i.e., larger cells and more cytoplasmic extensions compared with cells observed at 24 h. Most of the spread cells on the SLA and Mn-SLA surfaces were polygonal in shape, but cells on the Mn-SLA surface displayed a more accentuated cytoskeletal arrangement (actin filaments) and enhanced focal adhesions (vinculin expression) compared with the SLA surface (Figure 7).

**Figure 8:**
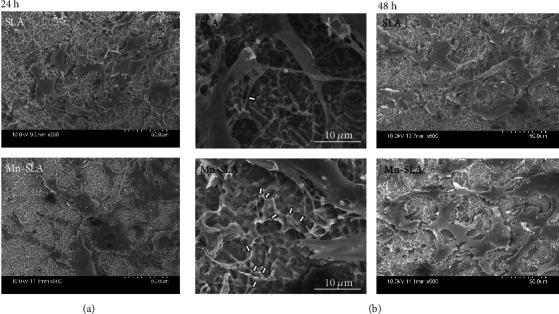
FE-SEM images showing the morphology of spread human mesenchymal stem cells on the investigated surfaces at 24 and 48 h of incubation. (a) FE-SEM images of spread cells at 24 h (at a magnification of ×600). (b) Higher magnification images showing the morphology of spread cells with lamellipodial projections (asterisk) and filopodial adhesions (arrow) at 24 h of incubation.

Together with the CLSM observation, the spread cell morphologies and the development of a fine attachment apparatus in MSCs adherent to the investigated samples were further evaluated by FE-SEM observation. Similar to the findings of CLSM, cells on the Mn-SLA surface displayed a more spread morphology at both 24 and 48 h (Figure 8). The cells grown on the Mn-SLA sample exhibited a more accentuated fine attachment apparatus, i.e., filopodial attachments (Figure 8(b)). Studies have demonstrated that vinculin is recruited and strongly activated at the adhesion site of the integrin-extracellular matrix complex at the cell interface [44].

Thus, the findings of a greater number of filopodial attachments, a concurrent increase in focal adhesions, and increased expression of critical adhesion related genes in cells grown on the Mn-SLA samples indicate that surface Mn modification enhances early cellular events in MSCs, events which are required for subsequently inducing osteogenesis-related intracellular signaling in the SLA implant surface.

### 3.5. Osteogenic Human MSC Differentiation Affected by Surface Mn Modification

#### 3.5.1. Osteogenesis-Related Gene Expression

**Figure 9:**
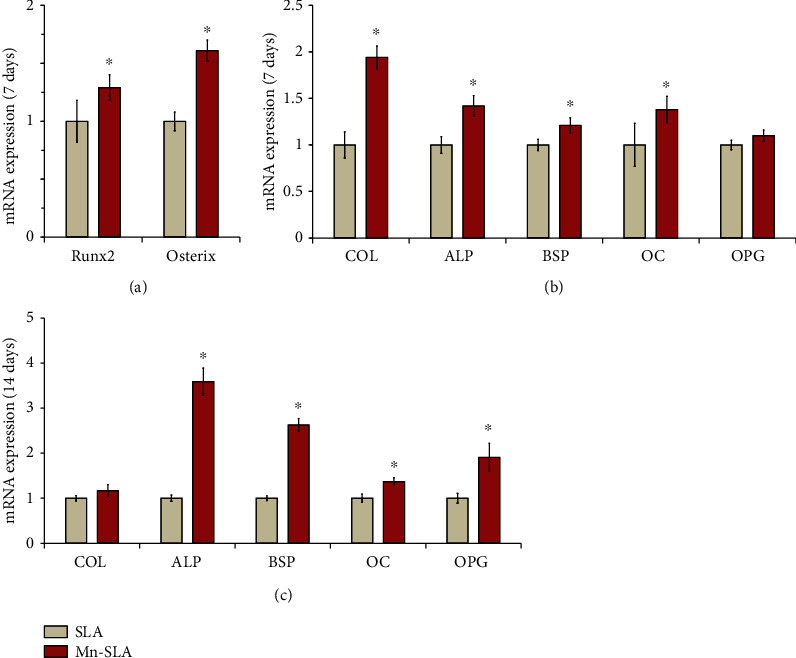
Quantitative real-time PCR analysis of the levels of mRNA for osteogenesis-related genes in human mesenchymal stem cells grown on the investigated surfaces at 7 and 14 days of culture. (a) mRNA levels of the transcription factor genes for osteogenic differentiation (Runx2 and osterix) at 7 days of culture. mRNA levels of the osteoblast phenotype genes (type I collagen (COL), ALP, bone sialoprotein (BSP), osteocalcin (OC), and osteoprotegerin (OPG)) at (b) 7 and (c) 14 days of culture. Values are the mean ± SD of three independent experiments. ^∗^*P* < 0.05 between two surfaces.

After assessing the early cell response evoked by surface Mn modification, we investigated the osteogenic capacity of Mn-incorporated SLA implant surface by assessing osteogenesis-related gene expression using real-time PCR analysis. We evaluated the mRNA expression levels of certain master regulator genes of osteoblast differentiation (Runx2 and osterix) in human MSCs at 7 days of culture. The mRNA expression of Runx2 and osterix was notably upregulated in human MSCs grown on the Mn-SLA surface compared with the unmodified SLA surface (*P* < 0.05; Figure 9(a)). It has been demonstrated that Runx2 is essential for the differentiation of MSCs into preosteoblasts [45], and osterix is an osteoblast-specific transcription factor for the differentiation of preosteoblasts into mature osteoblasts [45]. These findings indicate that surface Mn modification using wet chemical treatment is effective in initiating the first step in the osteogenesis-related cascade of MSCs, the upregulation of critical bone specific transcription factors, in the SLA-type implant surface.

We then assessed the mRNA expression of various osteoblast phenotype marker genes indicating the stages of early (COL and ALP) and intermediate (BSP) osteoblast differentiation and also mature osteoblasts (OC and OPG) at 7 and 14 days of incubation. The upregulated expression of transcription factor genes with surface Mn modification resulted in a concomitant increase of osteoblast phenotype gene expression (Figures 9(b) and 9(c)). At 7 days, human MSCs grown on the Mn-SLA surface displayed a greater expression level of marker genes for the early (COL, 1.9-fold; ALP, 1.4-fold), intermediate (BSP, 1.2-fold), and terminal (OC, 1.4-fold) stages of osteoblast differentiation than those on the SLA surface (*P* < 0.05; Figure 9(b)). At 14 days, cells grown on the Mn-SLA surface still displayed elevated osteoblast phenotype gene expression levels, except for COL (Figure 9(c)). ALP (3.6-fold) and BSP (2.6-fold) mRNA expression was significantly increased on the Mn-SLA surface (*P* < 0.05; Figure 9(c)). In addition, mRNA expression of mature osteoblast phenotype genes, i.e., OC (1.4-fold) and OPG (1.9-fold), was notably increased in MSCs by surface Mn modification (*P* < 0.05). The findings of increased OC and OPG mRNA expression on the Mn-SLA surface indicate that surface Mn modification promotes full differentiation of MSCs into mature osteoblasts in the microrough SLA-type Ti implants.

#### 3.5.2. ALP Activity and OC Protein Production

**Figure 10:**
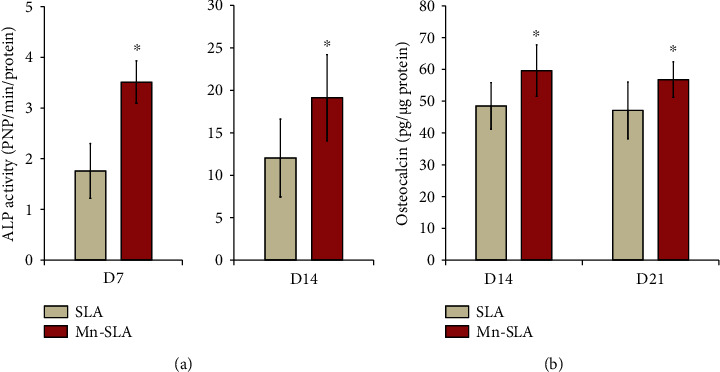
(a) ALP activity of human mesenchymal stem cells grown on the SLA and Mn-SLA surfaces at 7 and 14 days of culture. (b) ELISA results for the detection of the osteocalcin protein levels secreted into the culture media by human mesenchymal stem cells grown on the investigated surfaces at 14 and 21 days of culture. Values are the mean ± SD of three independent experiments. ^∗^*P* < 0.05 between two surfaces.

After evaluating osteogenic differentiation of human MSCs affected by surface Mn modification at the mRNA level, ALP and OC expression was further assessed also at the protein level. The ALP activity of human MSCs grown on the investigated samples is shown in Figure 10(a). Cells that were grown on the Mn-SLA surface exhibited significantly greater ALP activity than those on the SLA surface at 7 days of culture (*P* < 0.05). At 14 days, both surfaces displayed steeply increased ALP activity when compared with 7 days of incubation, but the Mn-SLA surface exhibited greater ALP activity than the SLA surface (*P* < 0.05; Figure 10(a)).

We then investigated whether surface Mn modification in fact promotes terminal osteoblast differentiation of MSCs when introduced into an SLA implant surface. For this purpose, we assessed OC protein production in adherent MSCs by ELISA. Human MSCs grown on the Mn-SLA surface secreted significantly greater OC protein into cell culture media at both 14 and 21 days of incubation compared with those on the SLA surface (*P* < 0.05; Figure 10(b)). These findings suggest that surface Mn modification by wet chemical treatment clearly promotes full differentiation of human MSCs into mature osteoblasts when applied to the SLA Ti implants.

In this study, we incorporated Mn ions into the surface of a clinical oral implant by wet chemical treatment. The resultant surface displayed improved corrosion resistance and encouraged human MSCs to express favorable osteogenesis-related cellular behaviors, including early cellular spreading and promotion of full osteoblast differentiation. Our findings indicate the efficacy of Mn as a potential candidate element for the surface chemistry alteration of modern oral implants in order to develop Ti implants with increased osteogenic capability and are in agreement with the results of other studies reporting that implant biocompatibility can be improved by surface Mn modification [11–13, 16–18].

However, there are wide discrepancies in the Mn ion concentration for inducing favorable osteogenesis-related cell functions [10–13, 17, 23]. Mn supplementation above 5.5 ppm significantly reduced the proliferative phase of the cell cycle (S and G2/M) and mRNA expression of COL and ALP in human MG63 osteoblastic cells, while 1.65 ppm did not exert any inhibitory effect on these cell functions [23]. Yu et al. [11] prepared Mn-containing titania coatings by microarc oxidation and assessed osteogenesis-related cell behavior in rat bone marrow MSCs. They showed that higher Mn content in the coatings induced more COL secretion and extracellular matrix mineralization, in which the Mn ion release from the high Mn content coating was approximately 1 ppm at 3 days of incubation [11]. On the other hand, osteogenic differentiation, including mRNA expression of osteoblast-related genes, ALP activity, and OC protein secretion, was enhanced by a relatively lower Mn ion release level (approximately 0.25 ppm at 3 days and 0.35 ppm at 10 days of incubation) in rat bone marrow MSCs grown on the high nitrogen stainless steel with 16% Mn content [17].

In contrast, osteogenic cell functions were enhanced at a much higher concentration of Mn ion release [13]. More Mn release (approximately 3 ppm at 3 days) from a modified Ti surface induced a better response from MC3T3-E1 cells, including proliferation, ALP activity, and mineralized nodule formation, than a Ti surface with a lower Mn release (2.5 ppm at 3 days) [13]. Mn ion release at 24 h of incubation was still higher than the Mn ion concentration exerting osteogenesis-promoting effects in other studies [11, 17, 23], including the present study (1.61 ppm for 24 h and 2.08 ppm for 3 days in the static test mode). Interestingly, osteogenesis-related gene expression was notably increased in rat bone marrow MSCs grown on the Mn-containing acid-etched Ti surface obtained by plasma ion implantation and doping even at a quite low Mn release (10 ppb at 24 h of incubation) [12], which is virtually the same level as the blood Mn content in adults (mean value, 9.3 ppb) [46] and quite a bit lower than the bone Mn content (mean value, 3.9 ppm) [47]. We suppose that various differences in the study test conditions, including the cell type (e.g., human or animal origin and the type of cell line), culture method (direct or indirect), surface features of the Ti samples, contributed to the diverse outcome. Therefore, further detailed studies of tightly controlled experimental design are needed to clearly elucidate the mode of action underlying the enhanced osteogenic capacity of surface Mn-containing Ti implants.

Considering the results from various reports on the beneficial effects of surface Mn-containing implantation materials, it does not seem to be realistic at present to determine so-called optimal concentration of released Mn ions from solid biomaterial surface to induce better bone healing effects. In addition, Mn ion release solely cannot be regarded as the mechanism underlying the enhanced osteogenic effect of surface Mn-modified Ti implants. The improved biological outcome obtained by Mn-incorporated Ti implants seems to be attributable to combined effects of additional critical factors such as the micro- and nanoscale surface topographies of implants. We suppose that surface nanostructure synergistically contributes to the enhanced osteogenic capacity of the Mn-SLA surface by increasing the surface area and providing better anchorage sites for early cell adhesion.

## 4. Conclusions

This study evaluated the surface characteristics and *in vitro* osteogenic capacity of surface Mn-modified microstructured Ti oral implants. We incorporated Mn ions into the surface of a currently used clinical oral implant, in this study an SLA implant surface, by wet chemical treatment. The corrosion resistance of resultant surface and osteogenesis-related cell functions modulated by Mn incorporation were evaluated by electrochemical measurements and examining the response of primary human MSCs. The wet chemical treatment produced a Mn-containing nanostructured crystalline TiO_2_ thin film on the SLA implant surface without any alteration of the original micron-scale complex surface topography. The treated surface exhibited improved corrosion resistance, sustained Mn ion release, and enhanced osteogenesis-related MSC function. The Mn-incorporated Ti oxide layer enhanced early cellular events, including spreading and focal adhesion formation, and promoted full differentiation of human MSCs into mature osteoblasts in the SLA implant surface. These results suggest that surface Mn modification using wet chemical treatment is an effective approach for producing a Ti surface with increased osteogenic capacity and favorable osseointegration interface stability in modern clinical oral implants.

## Figures and Tables

**Table 1:** Primer sequences for real-time polymerase chain reaction.

Gene	Forward primer sequence (5′-3′)	Reverse primer sequence (5′-3′)
Integrin *β*1	TCC AAC CTG ATC CTG TGT CC	GCT GGT GTG GTT GCT GGA AT
Integrin *β*3	TCC TCA TCA CCA TCC ACG A	TTA TCA GCC TGT GCC ACG A
RhoA	GAA GAG GCT GGA CTC GGA TT	TGT GGC AGA TAT CGA GGT GG
Talin	TCG CAG CAA TGT CTT GTC CT	CCA CCA TGA TCG TCT TCA CA
Vinculin	CCA AGG AGG TTG CCA AGC AG	CTG TGA AGG AGA CTG TGC GG
Runx2	CAG ACC AGC AGC ACT CCA TA	GAA CTG CTG TGG CTT CCA TC
Osterix	AGT TCA CCT GCC TGC TCT G	TCT GAC TGG CCT CCT CTT C
COL	CTG ACC TTC CTG CGC CTG AT	ATG GCT GCA CGA GTC ACA CC
ALP	CAT CAG GAC GCC TTG GAG AA	CGT CGA ACA TCT GAG TGG CT
BSP	CCA CCA CCG TTG AAT ACG AG	TAG CCA TCG TAG CCT TGT CC
OC	GGC AGC GAG GTA GTG AAG A	TGT GGT CAG CCA ACT CGT C
OPG	CGG CAA CAC AGC TCA CAA GA	ACC TTC GAG CAG CTT CGT AG
GAPDH	CCT CTG ACT TCA ACA GCG AC	TCC TCT TGT GCT CTT GCT GG

COL: type I collagen; ALP: alkaline phosphatase; BSP: bone sialoprotein; OC: osteocalcin; OPG: osteoprotegerin; GAPDH: glyceraldehydes-3-phosphate dehydrogenase.

**Table 2:** Chemical composition of investigated surfaces by X-ray photoelectron spectroscopy (atomic %).

Group	Ti2p	O1s	C1s	Mn2p	Al2p	N1s	Na1s
SLA	18.5	46.7	28.7		2.6	1.8	1.7
Mn-SLA	20.1	49.5	24	2.9	1.2	1.1	1.2

## Data Availability

Data are available on request.
